# MixClone: a mixture model for inferring tumor subclonal populations

**DOI:** 10.1186/1471-2164-16-S2-S1

**Published:** 2015-01-21

**Authors:** Yi Li, Xiaohui Xie

**Affiliations:** 1 Department of Computer Science, University of California, Irvine, CA 92697 US; 2 Institute for Genomics and Bioinformatics, University of California, Irvine, CA 92697 US; 3 Center for Machine Learning and Intelligent Systems, University of California, Irvine, CA 92697 US

**Keywords:** Cancer genomics, Subclonal inference, Whole genome sequencing, Somatic copy number alteration, Allele frequency, Mixture model

## Abstract

**Background:**

Tumor genomes are often highly heterogeneous, consisting of genomes from multiple subclonal types. Complete characterization of all subclonal types is a fundamental need in tumor genome analysis. With the advancement of next-generation sequencing, computational methods have recently been developed to infer tumor subclonal populations directly from cancer genome sequencing data. Most of these methods are based on sequence information from somatic point mutations, However, the accuracy of these algorithms depends crucially on the quality of the somatic mutations returned by variant calling algorithms, and usually requires a deep coverage to achieve a reasonable level of accuracy.

**Results:**

We describe a novel probabilistic mixture model, MixClone, for inferring the cellular prevalences of subclonal populations directly from whole genome sequencing of paired normal-tumor samples. MixClone integrates sequence information of somatic copy number alterations and allele frequencies within a unified probabilistic framework. We demonstrate the utility of the method using both simulated and real cancer sequencing datasets, and show that it significantly outperforms existing methods for inferring tumor subclonal populations. The MixClone package is written in Python and is publicly available at https://github.com/uci-cbcl/MixClone.

**Conclusions:**

The probabilistic mixture model proposed here provides a new framework for subclonal analysis based on cancer genome sequencing data. By applying the method to both simulated and real cancer sequencing data, we show that integrating sequence information from both somatic copy number alterations and allele frequencies can significantly improve the accuracy of inferring tumor subclonal populations.

## Background

Tumor genomes have been shown to present extensive cellular heterogeneity for decades since Nowell's original clonal theory for tumor progression [1]. Identifying tumor subclonal populations is important for both understanding the evolution of tumor cells, and for designing more effective treatments as pre-existing mutations occurring in some subclones could lead to drug resistance [2]. For example, a research in lymphocytic leukemia has shown links between the presences of driver mutations within subclones and adverse clinical outcomes [3].

With the advancement of next-generation sequencing (NGS) and launch of large-scale cancer genome sequencing projects [4], computational methods have recently been developed to infer tumor subclonal populations based on cancer genome sequencing data [5-9].

Most of these methods rely on sequence information from somatic point mutations, such as PyClone [5], EXPANDS [6], PhyloSub [7] and rec-BTP [8]. Methods in this category leverage the cluster pattern of allele frequencies at somatic point mutations to detect distinct subclonal populations. However, as the determination of somatic point mutations is imperfect and the inclusion of false-positives is unavoidable [10], deep sequencing with more than 100X coverage is often required for subclonal inferences with high sensitivity and specificity [5,7,8].

Other approaches utilizing the read depth information from genomic segments with somatic copy number alterations (SCNAs) to infer the cellular prevalences of subclonal populations have also been developed, such as THetA [9]. THetA explores all combinations of copy number changes across all segments to infer the most likely collection of subclonal populations [9]. However, with the copy number information alone, THetA suffers from the "identifiability problem", where distinct combinations of tumor purity and ploidy are able to explain the read depth information from SCNAs equally well [9]. Additionally, the running time of THetA scales exponentially with the number of genomic segments [9], and often takes a prohibitively long time to run under certain parameter settings.

In this article, we present a novel probabilistic mixture model, MixClone, to infer the cellular prevalences of subclonal populations. MixClone integrates both read depth information from genomic segments with SCNAs and allele frequency information from heterozygous single-nucleotide polymorphism (SNP) sites within a unified probabilistic framework. Such integrative framework has been shown to significantly improve the accuracy of tumor purity estimation in our previous work [11]. Here, we present that MixClone achieves two major advantages compared to the existing methods that (i) it does not require deep sequencing data, (ii) it resolves the identifiability problem. To demonstrate MixClone's utility, we conducted simulation studies and showed that it outperforms existing methods. We also applied MixClone on a breast cancer sequencing dataset [12], and showed that it was able to discover subclonal events not reported before.

## Methods

In this section, we introduce the generative mixture model of MixClone, which is an extension of our previous work on tumor purity estimation[11]. First, we introduce the notations for input data. Then, we describe the probabilistic models for sequence information of both SCNAs and allele frequencies. Finally, we combine these two types of data into a single likelihood model, and describe an algorithm to solve the model.

### Basic notations

The raw input data for MixClone are two aligned whole genome sequencing read sets of paired normal-tumor samples and a genome segmentation file based on the tumor sample. Following the notations from our previous work [11], we assume the tumor genome has been partitioned into *J *segments. We also assume there are *I_j _*heterozygous SNP sites within segment *j *in the corresponding normal genome, and use (*i*, *j*) to index SNP site *i *within segment *j*. For each SNP site (*i*, *j*) we define the A allele to be the reference allele and the B to be the alternative allele, with respect to the reference genome. We also use a superscript N to denote data from normal samples and superscript T to denote data from tumor samples. Overall, the observed data are summarized in the following notations [13]:

$b_{i\;j}^{N}$ = number of reads mapped to the B allele in the normal sample at site (*i*, *j*).

$d_{i\;j}^{N}$ = reads depth of the normal sample at site (*i*, *j*).

$D_{j}^{N}$ = total number of reads mapped to segment *j *of the normal sample.

The notations for the observed data from tumor samples are similarly defined, e.g. $D_{j}^{T}$ denotes total number of reads mapped to segment *j *of the tumor sample.

### Modeling SCNAs

Next, we describe the probabilistic model for SCNAs data. For each segment *j*, we define an allelic configuration *H_j _*to represent its underlying allele-specific copy number status. For example, if the absolute copy number of segment *j *is 2, then the compatible allelic configurations are PP, MM and PM, where P and M denotes the paternal and maternal allele of the tumor genome, respectively. Since PP and MM are not distinguishable based on sequence information alone as the reference human genome is not phased, we define the set of all possible allelic configuration as

(1) $$H_{j}\in H=\left\{0̸,\text{P/M},\text{PP/MM},\text{PM},\text{PPP/MMM},\text{PPM/PMM}\right\}$$

assuming the maximum copy number for each segment is 3. The corresponding copy number associated with each allelic configuration in  H is then

(2) $$n_{h}=\left\{0,1,2,2,3,3\right\}$$

MixClone allows the user to specify the maximum copy number and the default value is 6 in the released package [11]. We further assume there are *K *subclonal populations within the tumor sample, each of which has an associated cellular prevalence *ϕ_k _*∈ 0[1]. The subclonal type of each segment *j *is denoted as

(3) $$Z_{j}\in Z=\left\{1,2,\cdot \;\cdot \;\cdot \;,K\right\}$$

representing one of the *K *possible subclonal populations. Given the allelic configuration *H_j _*= *h *and the subclonal type *Z_j _*= *k*, the average copy number of segment *j *within the tumor sample, taking into account the subclonal cellular prevalence *ϕ_k_*, is

(4) $${\bar{C}}_{j}=\phi_{k}n_{h}+\left(1-\phi_{k}\right)2$$

Based on the Lander-Waterman model [14], the probability of sampling a read from a given segment *j *depends on three main factors: 1) its copy number, 2) its total genomic length, and 3) its mappability, which depends on factors such as repetitive sequence and GC content [9]. For each segment *j*, we associate a coefficient *θ_j _*to account for the effect of its mappability and genomic length. Thus the expected read counts mapped to segment *j*, which is denoted as *λ_j_*, is proportional to ${\bar{C}}_{j}\theta_{j}$. For example, for segment *x *and segment *y*, we have

(5) $$\frac{\lambda_{x}}{\lambda_{y}}=\frac{{\bar{C}}_{x}\theta_{x}}{{\bar{C}}_{y}\theta_{y}}$$

Because the mappability coefficients (*θ_j_*'s) matter only in a relative sense, we take $\theta_{x}\text{/}\theta_{y}=D_{x}^{N}\text{/}D_{y}^{N}$, as these segments should have the same sequence properties between the normal and tumor samples.

Additionally, to determine the absolute value of *λ_j_*, we curate a list of segments which contain no loss of heterozygosity according to their allele frequencies information. Based on the observed number of reads mapped to each segment, we further remove "outlier" segments from the list if their copy numbers are different from the bulk of the segments' copy numbers in the list. Finally, we call the remaining segments in the list as "baseline segments" and denote the set of these segments as *S*. We assume the allelic configurations of all the baseline segments are PM with copy number *n_s _*= 2. Other possible allelic configurations for baseline segments, which have equal copy numbers for each allele (e.g. *φ*, PPMM), are likely to be rare, and currently we do not model them. Then based on *n_s_*, we specify *λ_j _*as follows

(6) $$\lambda_{j}=\frac{1}{\left|S\right|}\;\underset{s\;\in \;S}{\sum }\frac{{\bar{C}}_{j}\theta_{j}}{n_{s}\theta_{s}}\;D_{s}^{T}$$

where $D_{s}^{T}$ denotes the number of reads mapped to segment *s *of the tumor sample.

Finally, we model the number of reads mapped to segment *j *in the tumor sample as a Poisson distribution, given *H_j _*and *Z_j_*

(7) $$D_{j}^{T}|H_{j},\;Z_{j}\sim \text{Poisson}\;\left(\lambda_{j}\right)$$

Details on curating the baseline segments are given in Supplementary, Additional file 1.

### Modeling allele frequencies

Next, we describe the probabilistic model used for allele frequencies of heterozygous SNP data. For each SNP site *i *within segment *j*, we denote its tumor genotype as *G_ij_*, which is selected from the set of all possible tumor genotypes up to a maximum copy number alteration, e.g.

(8) G={0̸,A,B,AA,AB,BB,AAA,AAB,ABB,BBB}

assuming the maximum copy number is 3. The corresponding B allele frequencies (BAF) for all the genotypes in G are

(9) $$\mu_{g}=\left\{\frac{1}{2},\epsilon ,1-\epsilon ,\epsilon ,\frac{1}{2},1-\epsilon ,\epsilon ,\frac{1}{3},\frac{2}{3},1-\epsilon \right\}$$

in which, *ε *≪ 1 is a small random deviation accounting for general sequencing errors. We choose *E *= 0.01, which is equivalent to a Phred quality of 20 [15].

Given the tumor genotype *G_ij _*= *g*, the allelic configuration *H_j _*= *h*, and the subclonal type *Z_j _*= *k*, the average BAF of site (*i*, *j*) within the tumor sample, taking into account the subclonal cellular prevalence *φ_k_*, is

(10) $${\bar{\mu }}_{ij}=\frac{\phi_{k}n_{h}\mu_{g}+\left(1-\phi_{k}\right)2\mu_{0}}{\phi_{k}n_{h}+\left(1-\phi_{k}\right)2}$$

in which *µ*_0 _= 0.5 is the BAF of heterozygous SNP sites in the normal sample. Finally, we model the distribution of the B allele count $b_{ij}^{T}$ at site (*i*, *j*) as a binomial distribution, given *G_ij _*, *H_j _*and *Z_j_*

(11) $$b_{ij}^{T}|d_{ij}^{T},G_{ij},H_{j},Z_{j}\sim \text{Binomial}\left(d_{ij}^{T},{\bar{\mu }}_{ij}\right)$$

### Combining SCNAs and allele frequencies

Now, we combine sequence information from both SCNAs and heterozygous SNP sites. For all the heterozygous SNP sites within the same segment, their genotypes should be consistent with the underlying allelic configuration of the segment. We model this consistency through a predefined conditional probability $Q_{gh}=\mathbb{P}\left(G_{ij}=g|H_{j}=h\right)$. If the genotype *g *is inconsistent with the allelic configuration *h*, e.g. AA is inconsistent with PM, we assign a small probability *σ *as *Q_gh_*, otherwise we assign equal probabilities to genotypes that are consistent with the allelic configuration.

Conditional on the underlying allelic configuration *H_j _*and subclonal type *Z_j_*, the probability of observing B allele read count $b_{ij}^{T}$ at site (*i*, *j*) is given as

(12) $$\mathbb{P}\left(b_{ij}^{T}|H_{j}=h,Z_{j}=k\right)=\underset{g\in G}{\sum }Q_{gh}\mathbb{P}\left(b_{ij}^{T}|G_{ij}=g,H_{j}=h,Z_{j}=k\right)$$

We assume that conditional on the allelic configuration *H_j _*, the B allele read counts ${\left\{b_{ij}^{T}\right\}}_{i=1}^{I_{j}}$ at different sites within the same segment *j *are independent of each other, and are also independent of the total read count $D_{j}^{T}$ of the segment. Then, the joint probability of observing the two types of read counts information of segment *j *is

(13) $$\begin{array}{c} \;\mathbb{P}\left(D_{j}^{T},{\left\{b_{ij}^{T}\right\}}_{i=1}^{I_{j}}|H_{j}=h,Z_{j}=k\right)\\ =\mathbb{P}\left(D_{j}^{T}|H_{j}=h,Z_{j}=k\right)\times \overset{I_{j}}{\underset{i=1}{\prod }}\underset{g\in G}{\sum }Qgh\mathbb{P}\left(b_{ij}^{T}|G_{ij}=g,H_{j}=h,Z_{j}=k\right) \end{array}$$

### Likelihood model

We have specified the joint distribution of the two types of read counts information of segment *j*. We then further model the allelic configuration *H_j _*and the subclonal type *Z_j _*of segment *j *as random variables that follow categorical distributions

(14) $$H_{j}|\rho_{j}\sim \text{Categorical}\left(\rho_{j}\right)$$

(15) $$Z_{j}|\pi \sim \text{Categorical}\left(\pi \right)$$

*ρ*_*j *_= (*ρ*_*j*∅_, ⋯, *ρ*_*j*PPM/PMM_), where $\rho_{jh}=\mathbb{P}\left(H_{j}=h\right)$ is the probability of observing *h *as the allelic configuration of segment *j*. *π *= (*π*_1_, ⋯, *π_K_*), where $\pi_{k}=\mathbb{P}\left(Z_{j}=k\right)$ is the probability of observing subclonal type *k *for all the segments. The model parameters Θ is defined as

(16) $$\Theta =\left({\left\{\rho_{j}\right\}}_{j=1}^{J},{\left\{\pi_{k}\right\}}_{k=1}^{K},{\left\{\phi_{k}\right\}}_{k=1}^{K}\right)$$

And the model likelihood of observing all the data is then

(17) $$\begin{array}{c} \;\mathbb{P}\left({\left\{D_{j}^{T}\right\}}_{j=1}^{J},{\left\{b_{ij}^{T}\right\}}_{i=1,j=1}^{I_{j,}J}|\Theta \right)\\ =\overset{J}{\underset{j=1}{\prod }}\overset{K}{\underset{k=1}{\sum }}\underset{h\in H}{\sum }\mathbb{P}\left(Z_{j}=k\right)\mathbb{P}\left(H_{j}=h\right)\mathbb{P}\left(D_{j}^{T}|H_{j}=h,Z_{j}=k\right)\\ \times \overset{Ij}{\underset{i=1}{\prod }}\underset{g\in G}{\sum }Q_{gh}\mathbb{P}\left(b_{ij}^{T}|G_{ij}=g,H_{j}=h,Z_{j}=k\right)\\ =\overset{J}{\underset{j=1}{\prod }}\overset{K}{\underset{k=1}{\sum }}\underset{h\in H}{\sum }\pi_{k}\rho_{jh}\frac{\lambda_{j}^{D_{j}^{T}}e^{-\lambda_{j}}}{D_{j}^{T}!}\\ \times \overset{I_{j}}{\underset{i=1}{\prod }}\underset{g\in G}{\sum }Q_{gh}\left(\begin{array}{c} d_{ij}^{T}\\ b_{ij}^{T} \end{array}\right){\bar{\mu }}_{ij}^{b_{ij}^{T}}\left(1-{\bar{\mu }}_{ij}\right)d_{ij}^{T}-b_{ij}^{T} \end{array}$$

We use Expectation-Maximization (EM) algorithm [16] to find the maximum likelihood estimation of Θ. The complete details of the EM updates are given in Supplementary, Additional file 1.

### Model selection

One of the key issues in subclonal analysis is to determine the number of subclonal populations *K*. PyClone and PhyloSub use posterior sampling methods to estimate *K *[5,7], while THetA requires users to specify *K *as an input [9]. Since the probabilistic model of MixClone is a generative mixture model, the model complexity and the corresponding log-likelihood increases as *K *increases. Therefore, we use a criterion based on the increase of the log-likelihood to select *K*. Practically, Mix-Clone allows the user to specify *K*. If *K *is not specified, MixClone runs the mixture model five times with different *K *in range of 1 to 5. We denote the log-likelihoods under the five different settings as ${\left\{L_{K}\right\}}_{K=1}^{5}$, and the total log-likelihood increase as

(18) $$\Delta =L_{5}-L_{1}$$

If |Δ*/L*_1_| < 0.01, which means the ratio of total log-likelihood increase is less than 0.01, MixClone predicts there is no subclonal event in the tumor sample and selects *K *= 1 as the number of subclonal populations. If |Δ */L*_1_| ≥ 0.01, MixClone further calculates another quantity

(19) $$\delta_{i}=|L_{i}-L_{1}|\Delta ,i\in \left[2,5\right]$$

which is the cumulative log-likelihood increase from *K *= 1 to *K *= *i *as a percentage regarding to the total increase Δ. If *δ_i _*≥ 0.9 and *δ*_*i*−1 _< 0.9, MixClone selects *K *= *i *as the number of subclonal populations.

In practice, we suggest users use this criterion as a heuristic guide when analyzing real data, and determine the number of subclonal populations in conjunction with regard to other external information.

### MixClone software package

**Figure 1:**
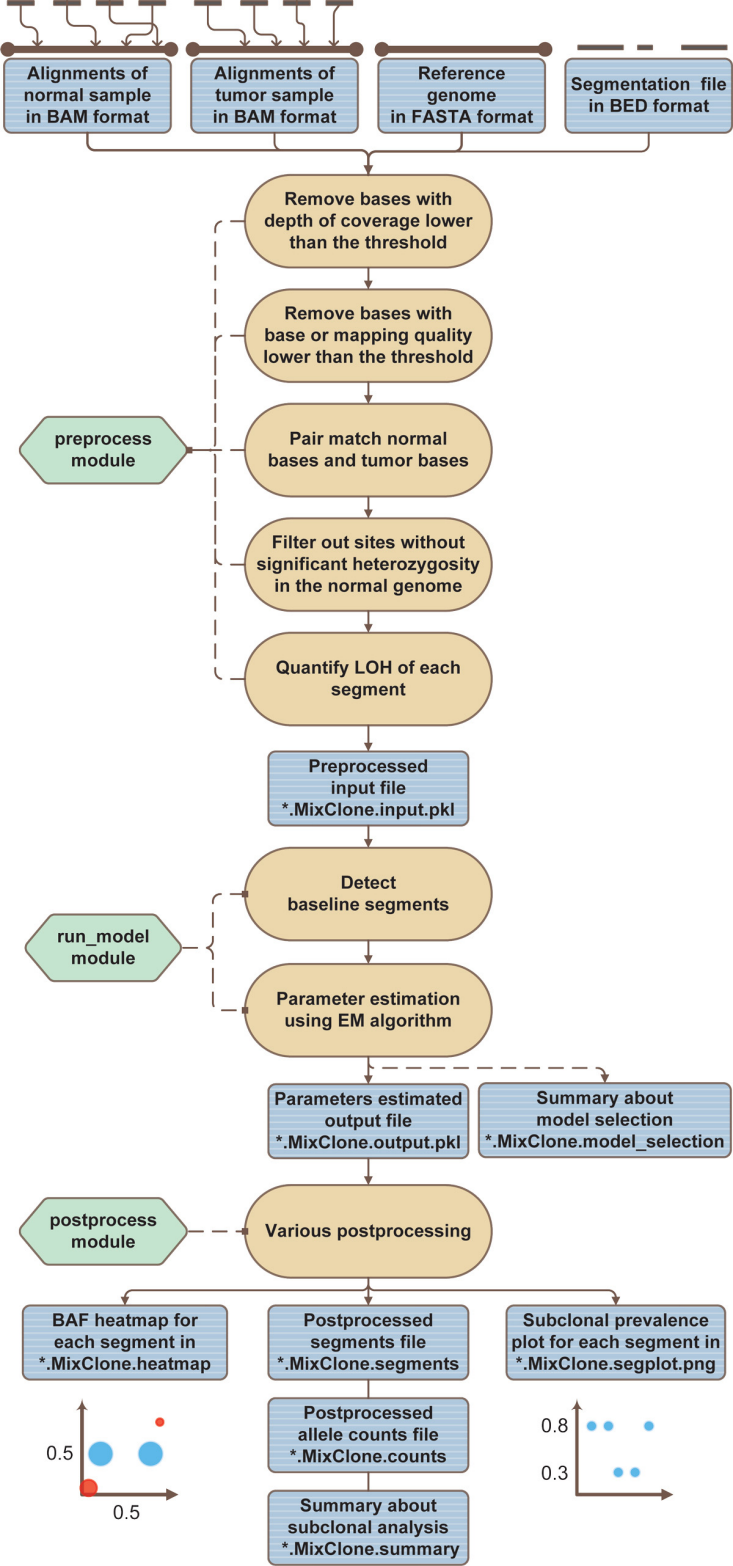
**The general workflow of MixClone**.

Figure 1 is the general workflow of MixClone. MixClone is a comprehensive software package, including subclonal cellular prevalences estimation, allelic configuration estimation, absolute copy number estimation and a few visualization tools. This package is implemented in Python and is built on top of the PyLOH package, previously released by us [11]. It also utilizes some features from the software package JointSNVMix [13], which have been explicitly indicated in the source code.

## Results

In this section, we evaluate the performance of MixClone on both simulated and real datasets and compare its performance with two published algorithms: (i) PyClone, a method based on somatic point mutations, and (ii) THetA, a method based on somatic copy number alterations.

### Results from simulated data

To generate simulation data, we simulated ten sets of NGS reads from chromosome 1 of artificial paired normal-tumor samples, each with 60X coverage. Heterozygous SNP sites from dbSNP [17] were inserted to the reference human genome to create the artificial normal genome. Both heterozygous SNP sites and somatic point mutations from [18] were inserted to the reference human genome to create artificial tumor genomes. Five of the artificial tumor genomes contain two subclonal populations and the other five contain three subclonal populations. Each artificial tumor genome was randomly assigned with segmentations, allelic configurations and subclonal cellular prevalences. We used segmentations based on both ground truth and BIC-seq [19] as the input for MixClone. We used ground truth somatic point mutation sites and copy numbers as the input for PyClone and THetA. Details on how reads were simulated and preprocessed are given in Supplementary, Additional file 1.

**Figure 2:**
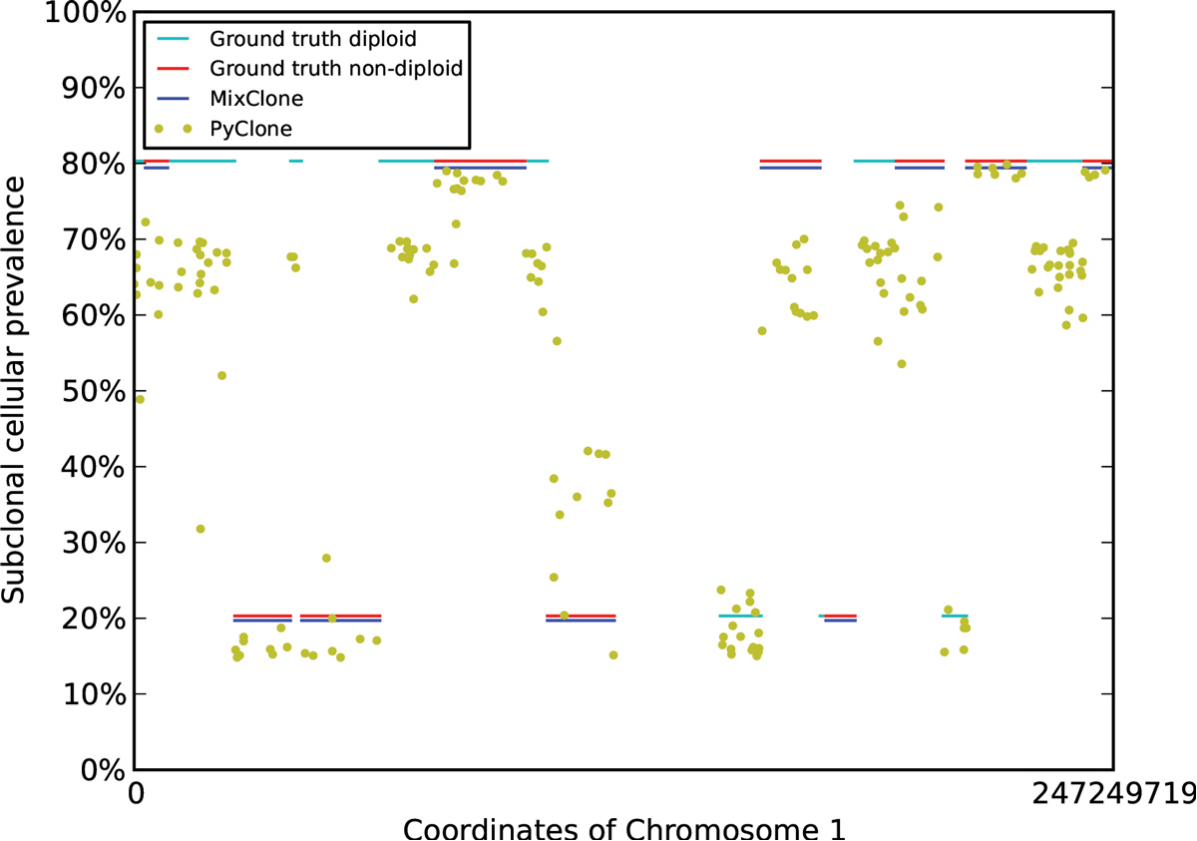
**Subclonal inference results by MixClone and PyClone on a simulated dataset with two subclonal populations**. The x-axis are the coordinates of Chromosome 1, and the y-axis are subclonal cellular prevalences. The blue horizontal bars represent the subclonal cellular prevalences estimated by MixClone based on non-diploid segments. Cyan and red horizontal bars represent the ground truth subclonal cellular prevalences of diploid and non-diploid segments. Yellow dots represent the subclonal cellular prevalences estimated by PyClone based on somatic point mutations.

MixClone is able to identify the correct subclonal populations for all the simulated datasets based on ground truth segmentations. Figure 2 shows the result of simulated dataset with two subclonal populations. MixClone also correctly estimates the subclonal cellular prevalences of all the segments with SCNAs except for one small segment in tumor genome case 4 with three subclonal populations. For results based on BIC-seq segmentations, MixClone still correctly estimates the subclonal cellular prevalences of the majority of the segments with SCNAs, except for those with copy-neutral loss of heterozygosity. This is likely due to the incorrect segmentations of BIC-seq, as BIC-seq relies on copy number changes and is unable to detect segments with copy-neutral loss of heterozygosity when they are adjacent to diploid segments. The complete results of all the simulated datasets based on both ground truth and BIC-seq segmentations are shown online through the github website associated with MixClone. As a comparison, we also run PyClone and THetA on the same datasets. We were unable to obtain THetA results after running it for more than 72 hours, likely due to its exponential scalability with the number of segments. In Figure 2, PyClone detects one of the two subclonal populations, whose ground truth cellular prevalence is 20%, but misestimates the other subclonal population, whose ground truth cellular prevalence is 80%, except for a few segments. The performance of MixClone on the other simulated datasets also significantly outperforms PyClone. One possible reason might be that the reads coverage of simulated datasets is not deep enough to support PyClone's non-parametric method [5], thus PyClone tends to report more subclonal populations due to the statistical variance.

### Results from breast cancer sequencing data

We also applied MixClone on a whole-genome breast cancer sequencing dataset [12]. The details on data preprocessing are described in Supplementary, Additional file 1.

**Figure 3:**
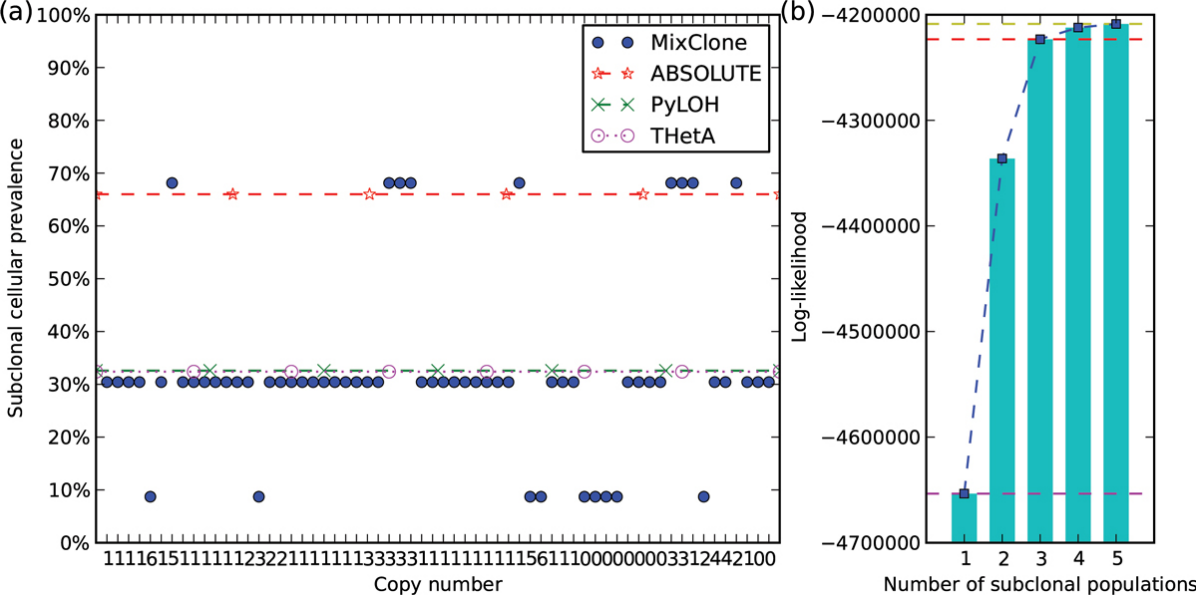
**Subclonal inference results of sample MB-116**. (a) The subclonal cellular prevalences estimated by MixClone, the tumor purities estimated by PyLOH, THetA [11], and the tumor purities estimated by ABSOLUTE [20] reported in [12] of sample MB-116. Each blue dot represents a segment. The x-axis is the estimated absolute copy number of the segment, and the y-axis is the estimated subclonal cellular prevalence of the segment. (b) The five log-likelihoods of MB-116 under different number of subclonal populations.

Figure 3a shows the subclonal inference results of sample MB-116. One estimated subclonal cellular prevalence 32% is consistent with the tumor purities estimated by PyLOH and THetA [11], and another estimated cellular prevalence 66% is consistent with the tumor purity estimated by ABSOLUTE [20] reported in [12].

Figure 3b shows the five log-likelihoods of MB-116 under different numbers of sub-clonal populations. The magenta, red and yellow curves represent the log-likelihoods corresponding to number 1, 3, and 5, respectively. Because the distance between the magenta and red curves (the cumulative log-likelihood increase from 1 to 3) is greater than 0.9 of the distance between the magenta and yellow curves (the total log-likelihood increase from 1 to 5), MixClone selected *K *= 3 as the number of subclonal populations for MB-116.

**Figure 4:**
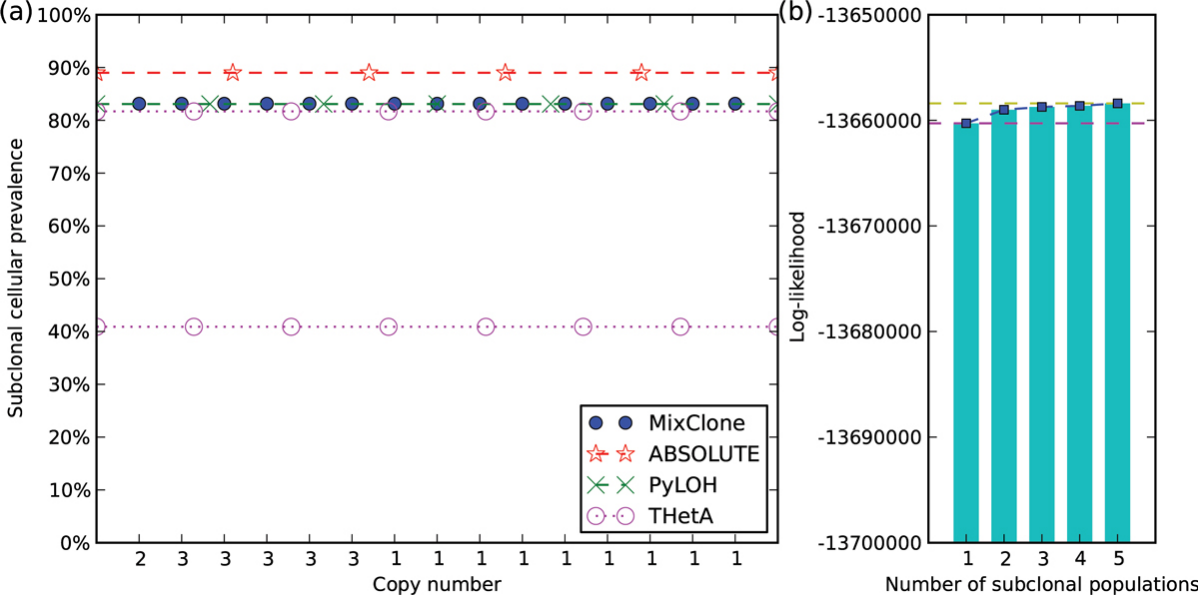
**Subclonal inference results of sample MB-106**. (a) The subclonal cellular prevalences estimated by MixClone, the tumor purities estimated by PyLOH, THetA [11], and the tumor purities estimated by ABSOLUTE [20] reported in [12] of sample MB-106. Each blue dot represents a segment. The x-axis is the estimated absolute copy number of the segment, and the y-axis is the estimated subclonal cellular prevalence of the segment. (b) The five log-likelihoods of MB-106 under different number of subclonal populations.

For samples without significant subclonal events, MixClone selected one as the number of subclonal populations, e.g. MB-106 (Figure 4). In Figure 4b, the ratio of total log-likelihood increase from 1 to 5 is 1.4 × 10^−4^, which is less than the threshold of 0.01. Therefore, MixClone selected *K *= 1 as the number of subclonal populations for MB-106. The estimated cellular prevalence of this single population is 83%, which is also consistent with the tumor purities estimated by PyLOH, ABSOLUTE and one result of THetA [11] (Figure 4a).

Besides MB-116, MixClone also detected significant subclonal events in MB-45 and MB-123. Results of MB-45 and MB-123 are given in Supplementary, Additional file 1.

## Discussion

In this article, we demonstrated MixClone's utility using whole genome sequencing data. However, most of the existing cancer genome sequencing data are from exome sequencing. An important future direction is to extend the current methodology to handle the exome sequencing data. Yet, extending MixClone to whole exome sequencing data is not trivial, as reads coverage on targeted exonic regions are no longer randomly distributed due to probe's variable efficiency [21]. Instead of Poisson distribution, using Gaussian distribution to model reads depth ratios between tumor and normal samples might be more appropriate to account for such additional variances, which has been demonstrated in whole exome sequencing based copy number analysis [21].

Another important future direction to extend MixClone is to implement joint analysis based on multiple samples, which is supported by PyClone and PhyloSub [5,7]. Multiple samples have been obtained for a single heterogeneous tumor tissue both temporally and spatially, and joint analysis based on these samples may reveal additional patterns of the history of tumor progression [5].

Currently, MixClone runs the subclonal analysis five times with different number of subclonal populations in range of 1 to 5 by default. In reality, larger numbers of subclonal populations may coexist within one tumor sample, but in this case some of the populations are very likely to share similar cellular prevalences. Since Mix-Clone defines different subclonal populations based on distinct cellular prevalences, those populations with similar cellular prevalences may not be differentiated by MixClone. To achieve finer resolution of subclonal populations, subclonal lineages information would be necessary to further differentiate each population in addition to cellular prevalences. And phylogenetic methods may be possible solutions to explicitly incorporate subclonal lineages information [7].

## Conclusions

In summary, we have developed a new method for inferring tumor subclonal populations by integrating sequence information gathered from SCNAs and heterozygous SNP sites. We showed that our method outperforms existing ones on simulation data, and applying it to a real breast cancer dataset is able to reveal new subclonal events not discovered before. Compared with existing methods, our method requires no additional deep sequencing of somatic point mutation sites.

## Competing interests

The authors declare that they have no competing interests.

## Authors' contributions

Designed the experiments: YL and XX; Performed the experiments: YL; Wrote the paper: YL and XX; All authors contributed to the analysis, and approved the paper.

## Supplementary Material

Additional file 1 **Complete details of (1) detecting heterozygous SNP sites, (2) curating the baseline segments, (3) the EM updates of MixClone, (4) reads simulation for simulated data and (5) reads preprocessing for both simulated data and breast cancer sequencing data**. Click here for file
