# Supplementary material for: MixClone: a mixture model for inferring tumor subclonal populations
Source: BMC Genomics. 2015 Jan 21;16(Suppl 2):S1. doi: 10.1186/1471-2164-16-S2-S1 (PMC4331709; doi:10.1186/1471-2164-16-S2-S1)
Supplement: Additional file 1 — Complete details of (1) detecting heterozygous SNP sites, (2) curating the baseline segments, (3) the EM updates of MixClone, (4) reads simulation for simulated data and (5) reads preprocessing for both simulated data and breast cancer sequencing data. [file 1471-2164-16-S2-S1-S1.pdf]

Supplementary for "MixClone: a mixture model for inferring tumor subclonal populations"

By Yi Li

1 Detecting heterozygous SNP sites

The probabilistic model of MixClone incorporates allele frequency information of each genomic segment by modelling the read counts information of the heterozygous SNP sites within each segment. To identify heterozygous SNP sites, we filter out sites without significant heterozygosity in the normal sample, which is defined as

$$b_{ij}^N / d_{ij}^N < 0.40 \texttt{ or } b_{ij}^N / d_{ij}^N > 0.60$$

2 Curating the baseline segments

To determine the absolute value of read counts mapped to each segment j, which is denoted as λ_j, we curate a list of segments which contain no loss of heterozygosity (LOH) according to their allele frequencies information. Specifically, we quantify the significance of LOH of each segment in the tumor sample, which is often resulted from somatic copy number alterations (SCNAs). The LOH significance of each segment provides additional information of the allelic configuration of the segment, e.g. segments with significant LOH must have unequal copy numbers for each allele. To quantify the LOH significance of each segment in the tumor sample, we calculate the fraction of LOH sites in the segment. We determine whether a site is LOH based on its one-tailed p-value of the binomial test

$$p_{ij}^T = \text{Binomial\_cdf}(min(d_{ij}^T - b_{ij}^T, b_{ij}^T) | d_{ij}^T, 0.5)$$

A site is defined as LOH if $p_{ij}^T < 0.025$. Then based on the fraction of LOH sites within each segment we assign an LOH status to the segment

$$\begin{array}{rlll} & \texttt{LOH}_{fraction} & < \texttt{LOH}_{max}, & \texttt{LOH}_{status} = \text{FALSE} \\ \texttt{LOH}_{min} \leq & \texttt{LOH}_{fraction} & < \texttt{LOH}_{max}, & \texttt{LOH}_{status} = \text{UNCERTAIN} \\ & \texttt{LOH}_{fraction} & \geq \texttt{LOH}_{max}, & \texttt{LOH}_{status} = \text{TRUE} \end{array}$$

We choose $\texttt{LOH}_{min} = 0.09, \texttt{LOH}_{max} = 0.11$ in practice.

Those segment with LOH status "FALSE" are curated as the initial list of baseline segments. We further remove "outlier" segments from the list if their copy numbers, which are correlated with the read depth ratios between the tumor and the normal samples, are different from the bulk of the segments in the list. The remaining segments are defined as the final set of baseline segments.

3 EM algorithm

We find the MLE of the model parameters using the Expectation-Maximization (EM) algorithm [1]. Since we have two sets of latent variables H_j, Z_j for segment j, we define two auxiliary variables ψ_{jh}, κ_{jk} to denote the conditional probability of the latent variable H_j, Z_j, conditional on all the observed data and the model parameters Θ.

$$\begin{aligned} \psi_{jh} &= \mathbb{P}(H_j = h | D_j^T, \{b_{ij}^T\}_{i=1}^{I_j}, \Theta) \\ &= \frac{\sum_{k=1}^K \pi_k \rho_{jh} \mathbb{P}(D_j^T, \{b_{ij}^T\}_{i=1}^{I_j} | H_j = h, Z_j = k)}{\sum_{k=1}^K \sum_{h \in \mathcal{H}} \pi_k \rho_{jh} \mathbb{P}(D_j^T, \{b_{ij}^T\}_{i=1}^{I_j} | H_j = h, Z_j = k)} \\ \kappa_{jk} &= \mathbb{P}(Z_j = k | D_j^T, \{b_{ij}^T\}_{i=1}^{I_j}, \Theta) \\ &= \frac{\sum_{h \in \mathcal{H}} \pi_k \rho_{jh} \mathbb{P}(D_j^T, \{b_{ij}^T\}_{i=1}^{I_j} | H_j = h, Z_j = k)}{\sum_{k=1}^K \sum_{h \in \mathcal{H}} \pi_k \rho_{jh} \mathbb{P}(D_j^T, \{b_{ij}^T\}_{i=1}^{I_j} | H_j = h, Z_j = k)} \end{aligned}$$

Then, the expected complete log-likelihood is specified as

$$\mathcal{Q} = \sum_{j=1}^J \sum_{k=1}^K \kappa_{jk} \sum_{h \in \mathcal{H}} \psi_{jh} \left\{ log\ \pi_k + log\ \rho_{jh} + \left[D_j^T log\ \lambda_j - \lambda_j - log\ D_j^T! \right] + \sum_{i=1}^{I_j} log \left[\sum_{g \in \mathcal{G}} Q_{gh} \binom{d_{ij}^T}{b_{ij}^T} \bar{\mu}_{ij}^{b_{ij}^T} (1 - \bar{\mu}_{ij})^{d_{ij}^T - b_{ij}^T} \right] \right\}$$

and the subsequent E-step and M-step of the EM algorithm can be derived as follows

E-step

We calculate the conditional probabilities of the latent variables given the observed data and previously estimated model parameters

$$\begin{aligned}\psi_{jh}^{(t+1)} &= \mathbb{P}(H_j = h|D_j^T, \{b_{ij}^T\}_{i=1}^{I_j}, \Theta^{(t)}) \\ \kappa_{jk}^{(t+1)} &= \mathbb{P}(Z_j = k|D_j^T, \{b_{ij}^T\}_{i=1}^{I_j}, \Theta^{(t)})\end{aligned}$$

M-step

We estimate the model parameters based on the conditional probabilities calculated in E-step. For ρ_j, the MLE is given by

$$\rho_{jh}^{(t+1)} = \psi_{jh}^{(t+1)}$$

For π_k, the MLE is given by

$$\pi_k^{(t+1)} = \frac{\sum_{j=1}^{J} \kappa_{jk}^{(t+1)}}{J}$$

For each subclonal cellular prevalence ϕ_k, ideally we need to take the derivative of $\mathcal{Q}$ in respect to each ϕ_k to find the value that maximize $\mathcal{Q}$ as the MLE. However, the solution of $\frac{\partial \mathcal{Q}}{\partial \phi_k} = 0$ is not in closed-form. Thus, we numerically search each ϕ_k to find the value that maximize $\mathcal{Q}$. Additionally, the computation for the binomial distribution part of $\mathcal{Q}$, which accounts for the sequence information from heterozygous SNP sites, is time-consuming when applying iteratively numerical search. However, the log-likelihood contribution of the binomial distribution part to $\mathcal{Q}$ is negligible, comparing the to the Poisson distribution part, which accounts for the sequence information from SCNAs. So practically, we numerically search the value of each ϕ_k that only maximize the Poisson distribution part of $\mathcal{Q}$, denoted as $\mathcal{Q}_{\texttt{SCNAs}}$, as the MLE for ϕ_k, which is specified as

$$\mathcal{Q}_{\texttt{SCNAs}} = \sum_{j=1}^{J}\sum_{k=1}^{K} \kappa_{jk} \sum_{h\in\mathcal{H}} \psi_{jh} \left\{log\ \pi_k + log\ \rho_{jh} + \left[D_j^T log\ \lambda_j - \lambda_j - log\ D_j^T!\right]\right\}$$

4 Sequencing data simulation

We used the method described in [2] to simulate reads from artificial paired normal-tumor samples. First, we created the genomic sequences of the artificial normal genome and tumor genome. We inserted SNP sites into the sequence of the reference genome NCBI Build 36.1(hg18) to generate the sequences of the artificial normal genome with respect to the two alleles. Based on the SNP frequency observed in the human population (one SNP every 1.9kilobases [3]), we randomly selected 10% SNP sites of dbSNP 132 [4] for each allele, and substituted the base of the reference sequence with the variant base of each SNP site. To generate the sequences of the two alleles of the artificial tumor genome, we inserted the somatic point mutation sites reported in PR2832 [5] into the artificial normal genome, and substituted the base of the artificial normal genome with the variant base of each somatic point mutation site.

Next, based on the genomic sequences of the artificial tumor genome, we created ten heterogeneous tumor genomes that differ in their segmentations, allelic configurations and subclonal cellular prevalences. Among the ten heterogeneous tumor genomes, five of them contain two subclonal populations and the other five contain three subclonal populations. We only used chromosome 1 for all the simulated datasets to reduce computational time. We randomly segmented chromosome 1 (excluding the centromere, heterochromatin and 1q21.1) into 20 segments for tumor genomes with two subclonal populations and 30 segments for tumor genomes with three subclonal populations. For one half of the segments of each tumor genome, we assigned their allelic configuration as `PM`, as we assume the bulk of the baseline segments are diploid. For the other half of the segments, we randomly assigned them non-doploid allelic configurations with respect to both alleles based on a prior distribution, which is specified according to the edit distance between the non-doploid allelic configuration and the diploid allelic configuration `PM`. Specifically, we denote the edit distance between allelic configuration h and `PM` as d_h, e.g. the edit distance between $h =$ `PPMM` and `PM` is 2. Large edit distance is less likely to happen as it represents large somatic copy number alterations. Therefore, the probability of sampling allelic configuration h for a given segment j is specified as

$$\mathbb{P}(H_j = h) = \frac{\alpha^{d_h}}{\sum_{h\in\mathcal{H}} \alpha^{d_h}}$$

in which $\alpha < 1$ is a shrinkage coefficient to make $\mathbb{P}(H_j = h)$ decrease exponentially with the increase of the edit distance d_h. We choose $\alpha = 0.5$ in practice, and the set of allelic configurations $\mathcal{H}$ was formulated with maximum copy number 4 for all the simulated datasets.

Finally, for each subclonal population within each tumor genome, we randomly picked its cellular prevalence from the set $[0.2, 0.3, 0.4, 0.5, 0.6, 0.7, 0.8, 0.9]$, and each cellular prevalence is at least 0.2 away from other cellular prevalences within the same tumor genome. We uniformly sampled reads within each segment, assuming the sequencing process has no error and all the segments share the same mappability. Thus, the factors that affect the probability of sampling a read from a specific segment are only its copy number, subclonal cellular prevalence and genomic length. We simulated 150 million reads of single-end and 100-bp length for each read set, which is roughly 60X coverage for chromosome 1.

5 Sequencing data preprocessing

NCBI Build 36.1(hg18) was used as the reference genome for all the datasets. We used bowtie-0.12.8 [6] with the option "-n 3 -k 2" to align the reads of simulated datasets. We used BWA-0.7.5a [7] with the default setting to align the reads of the breast cancer datasets. We used segmentations based on both ground truth and BIC-seq-1.2.1 [8] for simulated datasets, and segmentations based on BIC-seq for the breast cancer datasets. For simulated datasets with segmentations based on BIC-seq, we used segments with genomic length longer than 1 million bp as the input. We used SAMtools-0.1.18 [9] to convert reads alignment from SAM format to BAM format with the option "-Sbh -q 1" and also eliminate duplicates. We used GATK-2.4-7 [10] for base quality score recalibration and indel realignment with dbSNP 132 [4] for the breast cancer datasets. For more details about the usage of the breast cancer datasets, please refer to [2].

6 Parameter setting details

6.1 MixClone details

We used "–max_copynumber 4" for the simulated datasets, and "–max_copynumber 6" for the breast cancer datasets.

6.2 PyClone details

We used the beta-binomial model of PyClone with the default setting to run all the analyses on the simulated datasets.

7 Supplementary figure 1-2

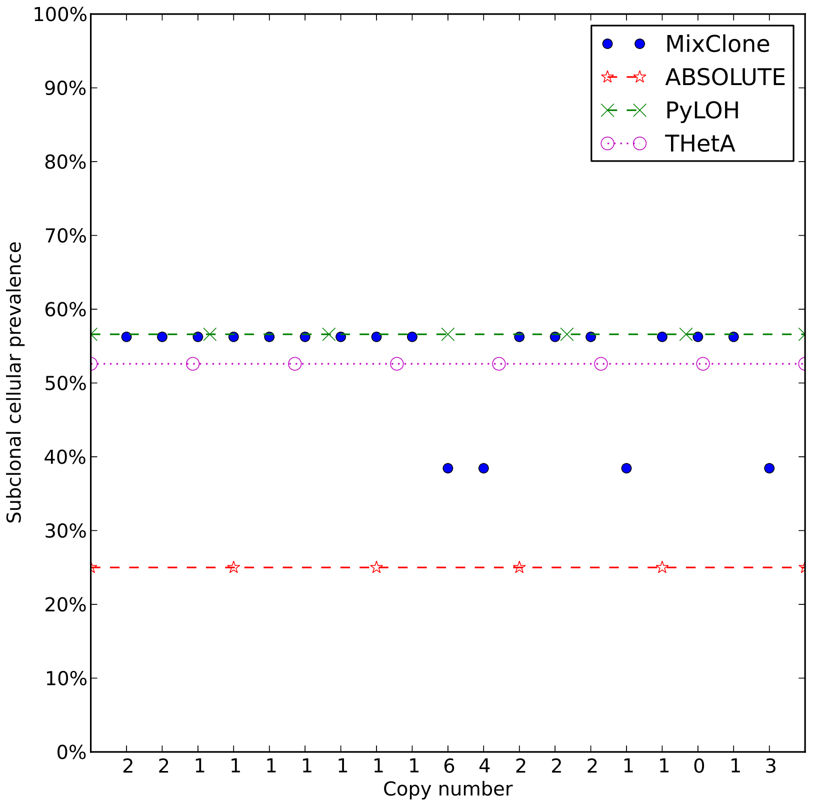

Figure S1: The subclonal cellular prevalences estimated by MixClone, the tumor purities estimated by PyLOH, THetA [2], and the tumor purities estimated by ABSOLUTE [11] reported in [12] of sample MB-45.

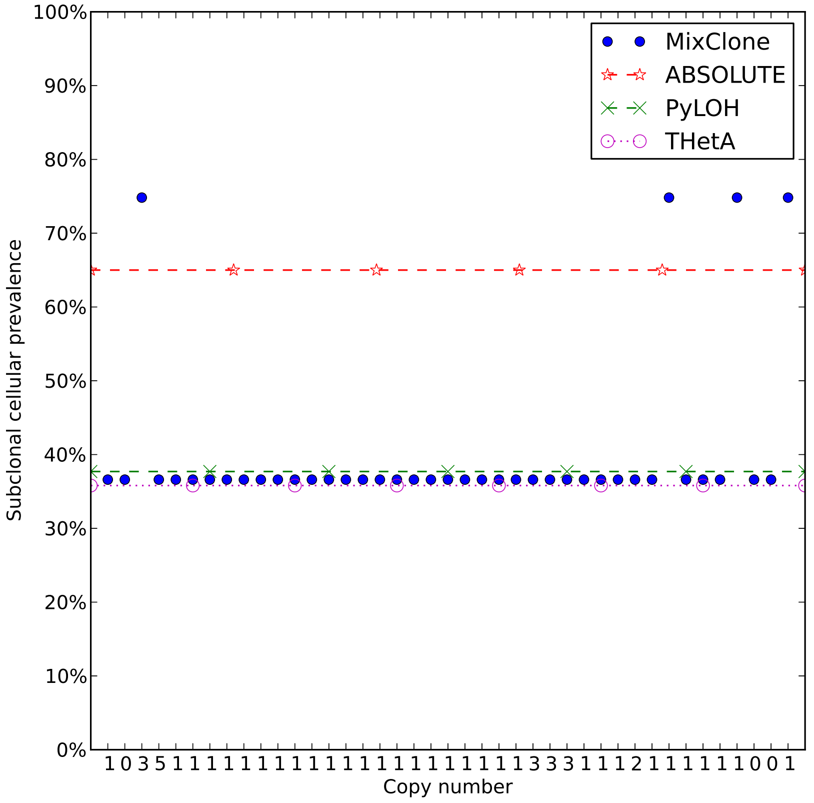

Figure S2: The subclonal cellular prevalences estimated by MixClone, the tumor purities estimated by PyLOH, THetA [2], and the tumor purities estimated by ABSOLUTE [11] reported in [12] of sample MB-123.

References

[1] Arthur P Dempster, Nan M Laird, and Donald B Rubin. Maximum likelihood from incomplete data via the em algorithm. *Journal of the Royal Statistical Society. Series B (Methodological)*, pages 1–38, 1977.

[2] Yi Li and Xiaohui Xie. Deconvolving tumor purity and ploidy by integrating copy number alterations and loss of heterozygosity. *Bioinformatics*, page btu174, 2014.

[3] Ravi Sachidanandam, David Weissman, Steven C Schmidt, Jerzy M Kakol, Lincoln D Stein, Gabor Marth, Steve Sherry, James C Mullikin, Beverley J Mortimore, David L Willey, et al. A map of human genome sequence variation containing 1.42 million single nucleotide polymorphisms. *Nature*, 409(6822):928–933, 2001.

[4] Stephen T Sherry, M-H Ward, M Kholodov, J Baker, Lon Phan, Elizabeth M Smigielski, and Karl Sirotkin. dbsnp: the ncbi database of genetic variation. *Nucleic acids research*, 29(1):308–311, 2001.

[5] Michael F Berger, Michael S Lawrence, Francesca Demichelis, Yotam Drier, Kristian Cibulskis, Andrey Y Sivachenko, Andrea Sboner, Raquel Esgueva, Dorothee Pflueger, Carrie Sougnez, et al. The genomic complexity of primary human prostate cancer. *Nature*, 470(7333):214–220, 2011.

[6] Ben Langmead, Cole Trapnell, Mihai Pop, Steven L Salzberg, et al. Ultrafast and memory-efficient alignment of short dna sequences to the human genome. *Genome Biol*, 10(3):R25, 2009.

[7] Heng Li and Richard Durbin. Fast and accurate short read alignment with burrows–wheeler transform. *Bioinformatics*, 25(14):1754–1760, 2009.

[8] Ruibin Xi, Angela G Hadjipanayis, Lovelace J Luquette, Tae-Min Kim, Eunjung Lee, Jianhua Zhang, Mark D Johnson, Donna M Muzny, David A Wheeler, Richard A Gibbs, et al. Copy number variation detection in whole-genome sequencing data using the bayesian information criterion. *Proceedings of the National Academy of Sciences*, 108(46):E1128–E1136, 2011.

[9] Heng Li, Bob Handsaker, Alec Wysoker, Tim Fennell, Jue Ruan, Nils Homer, Gabor Marth, Goncalo Abecasis, Richard Durbin, et al. The sequence alignment/map format and samtools. *Bioinformatics*, 25(16):2078–2079, 2009.

[10] Mark A DePristo, Eric Banks, Ryan Poplin, Kiran V Garimella, Jared R Maguire, Christopher Hartl, Anthony A Philippakis, Guillermo del Angel, Manuel A Rivas, Matt Hanna, et al. A framework for variation discovery and genotyping using next-generation dna sequencing data. *Nature genetics*, 43(5):491–498, 2011.

[11] Scott L Carter, Kristian Cibulskis, Elena Helman, Aaron McKenna, Hui Shen, Travis Zack, Peter W Laird, Robert C Onofrio, Wendy Winckler, Barbara A Weir, et al. Absolute quantification of somatic dna alterations in human cancer. *Nature biotechnology*, 30(5):413–421, 2012.

[12] Shantanu Banerji, Kristian Cibulskis, Claudia Rangel-Escareno, Kristin K Brown, Scott L Carter, Abbie M Frederick, Michael S Lawrence, Andrey Y Sivachenko, Carrie Sougnez, Lihua Zou, et al. Sequence analysis of mutations and translocations across breast cancer subtypes. *Nature*, 486(7403):405–409, 2012.
